# Materials and Techniques for Splinting Scan Bodies: A Scoping Review

**DOI:** 10.3390/ma19040664

**Published:** 2026-02-09

**Authors:** Aspasia Pachiou, Ioulianos Rachiotis, Alexis Ioannidis, Pune N. Paqué, Ronald E. Jung, Christos Rahiotis

**Affiliations:** 1 Clinic of Reconstructive Dentistry, Center for Dental Medicine, University of Zurich, Plattenstrasse 11, 8032 Zurich, Switzerland; aspasia.pachiou@zzm.uzh.ch (A.P.); alexis.ioannidis@zzm.uzh.ch (A.I.); punenina.paque@zzm.uzh.ch (P.N.P.); ronald.jung@zzm.uzh.ch (R.E.J.); 2 Dental School, National and Kapodistrian University of Athens, Thivon 2, 11527 Athens, Greece; sdn2100077@uoa.gr; 3 Department of Operative Dentistry, Dental School, National and Kapodistrian University of Athens, Thivon 2, 11527 Athens, Greece

**Keywords:** intraoral scan body, digital implant impressions, splinting techniques, auxiliary geometric devices, full-arch implant scanning, intraoral scanners

## Abstract

**Background:** Digital implant impressions using intraoral scanners are increasingly adopted; however, their accuracy remains challenging in complete-arch and extended edentulous scenarios due to limited anatomical reference points and cumulative stitching errors. Various splinting techniques, scan-body modifications, and auxiliary geometric devices have been proposed to enhance digital accuracy, yet the available evidence is highly heterogeneous and lacks comprehensive synthesis. **Methods:** This scoping review was conducted according to PRISMA-ScR guidelines. A systematic search of PubMed/MEDLINE, Embase, Scopus, and Web of Science databases identified studies evaluating materials, designs, or techniques intended to splint, stabilize, or geometrically augment intraoral scan bodies in digital implant workflows. In vitro, clinical, and mixed-design studies were included. Data were extracted descriptively and synthesized narratively. **Results:** Seventy-three studies met the inclusion criteria, the majority of which were in vitro investigations focused on fully edentulous arches. Splinting strategies included direct resin-based connections, rigid or semi-rigid auxiliary geometric devices, modified scan bodies with extensional geometries, and artificial landmarks. Most studies reported improved trueness, precision, or scanning efficiency when rigid or geometrically enriched devices were used, particularly in long-span or angulated implant configurations. However, flexible or optically interfering splints occasionally reduced accuracy, and outcomes were strongly scanner-dependent. **Conclusions:** Splinting and auxiliary scanning strategies generally enhance the accuracy of complete-arch digital implant impressions, especially when rigid, well-engineered, or geometrically complex designs are employed. Modified scan bodies and calibrated auxiliary devices appear particularly promising, while flexible splints may be counterproductive. Standardized protocols and further in vivo validation are required to optimize digital implant workflows.

## 1. Introduction

Implant-supported restorations have emerged as a treatment option and are currently considered a highly predictable and widely used modality for replacing missing teeth [1]. Regardless of the impression technique employed, the accurate transfer of the three-dimensional implant position is mandatory to obtain implant-fixed restorations with an adequate passive fit [2,3,4,5]. Misfits caused by inaccuracies in the impression process can lead to mechanical complications, such as screw loosening, fractures, and increased stress on the peri-implant bone, ultimately compromising long-term clinical outcomes [6,7].

To address the challenges of traditional methods, digital workflows have emerged, with intraoral scanners (IOSs) and CAD/CAM technologies becoming integral components of modern practice [8,9]. By eliminating traditional impression materials, these digital techniques reduce patient discomfort and minimize errors associated with material shrinkage or expansion [8]. Essential to this workflow are intraoral scan bodies (ISBs), which are scannable transfer abutments required to digitize the relative virtual position of the implant platform [10,11,12].

Studies have consistently shown that the trueness of IOS measurements is clinically acceptable for short edentulous spans or single-unit implant-supported prostheses [13,14]. However, digital scans are less accurate in complete edentulous arches [15,16]. This inaccuracy arises because common reference points in edentulous arches are limited, leading to incorrect image stitching or the misinterpretation of data as redundant [16,17]. Furthermore, the lengthy span between implants in full-arch cases exacerbates these stitching errors, as slight inaccuracies in aligning overlapping scans accumulate over the arch [18,19]. Due to these limitations in digital acquisition, the conventional open-tray implant impression with splinted impression copings remains the standard for complete-arch implant impressions, with reported high accuracy [20,21].

To enhance the accuracy of digital workflows for full-arch cases, new strategies have been proposed to overcome the limitations of standard protocols. A logical method to improve scanning accuracy is to increase the number of reference points by splinting scan bodies or using specific auxiliary devices; another approach is to place additional markers between implants or to use horizontal scan bodies to enhance geometric referencing [20,21,22,23,24,25]. These auxiliary geometric devices (AGDs) and splinting techniques aim to enhance scan body stability and improve alignment during scanning, potentially reducing stitching errors and improving the overall accuracy of the digital impression [26,27].

Despite the growing body of literature on digital implant impressions in edentulous arches, a clear clinical decision-making framework remains lacking. Current studies investigate heterogeneous strategies that can be broadly classified as (i) splinting approaches, which physically connect scan bodies to enhance stability; (ii) scan-body design modifications, which alter geometry to improve optical recognition; and (iii) auxiliary geometric devices, which add independent reference structures. Although all aim to reduce cumulative stitching errors, their clinical indications, complexity, and level of supporting evidence differ substantially, complicating clinical selection.

Given the diversity of interventions, study designs, outcome measures, and scanner technologies, a scoping review was deemed the most appropriate approach to map and categorize the available evidence rather than to compare outcomes or infer superiority. Accordingly, this review aims to clarify the conceptual landscape of these strategies, identify gaps in the literature, and distinguish between approaches supported primarily by in vitro data and those with emerging clinical evidence, thereby supporting clinical decision-making and guiding future standardized research.

## 2. Materials and Methods

### 2.1. Protocol and Reporting

This scoping review was designed and conducted according to the Preferred Reporting Items for Systematic Reviews and Meta-Analyses extension for Scoping Reviews (PRISMA-ScR) [28], ensuring transparent and structured methodology across all review stages. The protocol was prospectively registered in the Open Science Framework (OSF; Registration DOI: 10.17605/OSF.IO/STH42) on 5 November 2025, before data extraction, to prevent methodological deviations and enhance reproducibility.

The review aimed to systematically map the available evidence on materials, designs, and techniques used for splinting intraoral scan bodies (ISBs) during complete-arch and partial-arch digital implant impressions.

The Population–Concept–Context (PCC) framework guided the formulation of the research question:

- **Population:** Edentulous or partially edentulous patients, or in vitro models with implant-supported restorations.
- **Concept:** Any technique, material, device, modification, or scan-body geometry intended to enhance the accuracy of intraoral scan body–based digital impressions (Splinting materials and assemblies, auxiliary geometric devices (AGDs), calibration frameworks, Artificial markers/reference landmarks, Horizontal or modified scan bodies, custom 3D-printed attachments or scan-body enhancements and modular, prefabricated, or denture-like stabilizing structures.
- **Context:** Digital implant workflows in clinical and laboratory settings, including in vitro, in vivo, and clinical studies.

### 2.2. Eligibility Criteria

**Inclusion criteria were defined as follows:**

- Studies evaluating any splinting method, auxiliary device, geometric attachment, artificial marker or landmark, horizontal or modified scan body, or any material or technique designed to stabilize, augment, or enhance the geometric referencing of intraoral scan bodies during digital scanning.
- Accepted study types: in vitro studies, clinical trials, observational studies, case series with quantitative assessment, and technical reports containing measurable outcomes.
- Studies using either direct-to-implant ISBs or multi-unit abutment ISBs.
- Full-arch or partial-arch implant scenarios.
- Articles published in English in peer-reviewed journals.

**Exclusion criteria included:**

- Studies evaluating ISBs without any splinting or stabilizing method, unless serving as a control.
- Review articles, conference abstracts, expert opinions, case reports/techniques without measurable outcomes.
- Studies that solely assessed conventional impression splinting without digital comparison.
- Animal studies.

Technical reports were included only when they provided quantitative or measurable outcomes related to digital impression accuracy; reports lacking such data were excluded, as they did not allow meaningful synthesis within the scope of this review.

Measurable outcomes were defined as quantitative parameters related to digital impression performance, including trueness, precision, linear or angular deviation, RMS error, passive fit, misfit values, or scanning time. In borderline cases where outcome reporting was unclear, studies were included only if numerical data or reproducible quantitative comparisons were provided; otherwise, they were excluded by consensus between reviewers.

### 2.3. Information Sources and Search Strategy

A comprehensive electronic search was conducted across PubMed/MEDLINE, Embase, and Scopus to identify eligible studies involving splinting approaches for ISBs. The search strategies combined controlled vocabulary and free-text terms relating to:

- intraoral scan bodies and scanning abutments,
- splinting techniques (e.g., floss-resin splints, auxiliary geometric devices, verification frameworks),
- digital implant impressions, and
- CAD/CAM workflows.

Search terms were adapted for each database (Appendix A). The search strategy was finalized on 15 October 2025, prior to registration (5 November 2025); the database searches were executed, and screening was initiated only after registration. To maximize completeness, reference lists of included studies and related reviews were also manually screened.

### 2.4. Study Selection

Study selection occurred in two phases:

- Title and abstract screening were performed independently by two reviewers (A.P., I.R.) to exclude clearly irrelevant articles.
- Full-text assessment was carried out for potentially eligible studies, applying the predefined inclusion and exclusion criteria.

**Figure 1:**
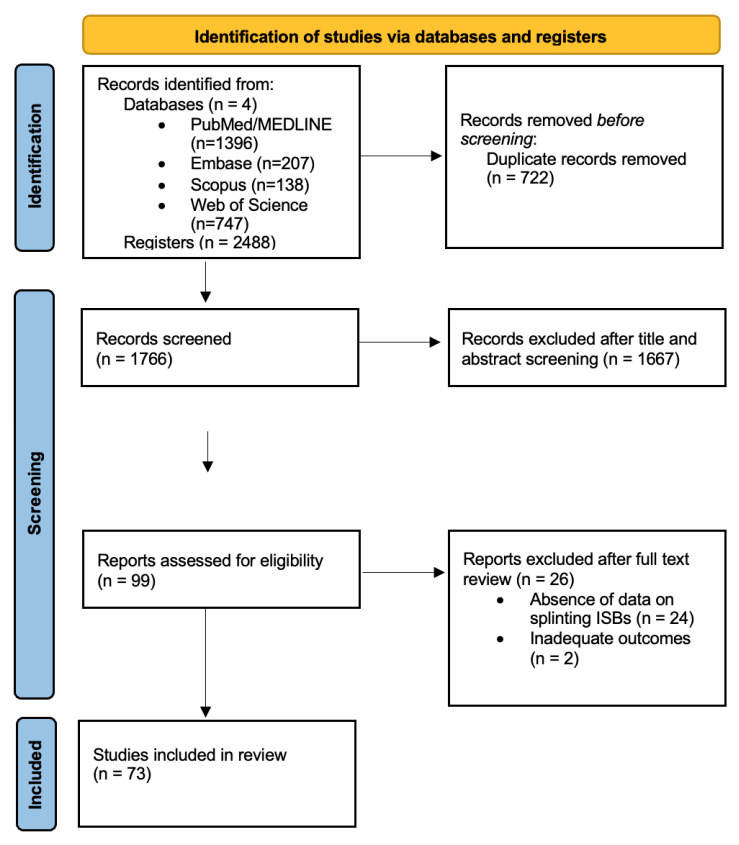
PRISMA 2020 flow diagram.

Any discrepancies between reviewers were resolved through discussion; when consensus was not achieved, a third reviewer (C.R.) adjudicated. The complete selection process is illustrated in the PRISMA flow diagram (Figure 1).

### 2.5. Data Extraction

A structured extraction form was created specifically for this review, drawing on the different experimental and clinical variables reported in the literature on ISB splinting techniques. Reference management and screening were performed using Rayyan (Qatar Computing Research Institute). The following information was collected from each study:

- Author(s)
- Publication year
- Study design (in vitro or clinical)
- Jaw or model type
- Edentulism pattern
- Number of implants
- ISB type
- Splinting material or device used (e.g., floss/resin, custom geometric aids, prefabricated bars, modular frameworks, dental floss with resin, 3D-printed attachments)
- Configuration of splinting (full-arch vs. non-continuous)
- Scanner type
- Control groups (e.g., non-splinted IOS, conventional impressions, laboratory scans)
- Key quantitative outcomes for trueness, precision, angular deviation, scanning time, and misfit values.

Data extraction was performed independently by two reviewers (A.P., I.R.), with disagreements resolved by consensus or by a third reviewer’s intervention (C.R.).

### 2.6. Data Synthesis

The extracted data were synthesized using a descriptive and narrative approach, consistent with the objectives and methodological framework of a scoping review. Given the substantial heterogeneity across included studies—particularly regarding study design, implant systems, scan body configurations, splinting materials, auxiliary devices, intraoral scanner technologies, and outcome metrics—a quantitative synthesis was not appropriate.

Studies were grouped and mapped according to key conceptual domains, including:

- type of splinting strategy or auxiliary scanning aid (direct splinting, auxiliary geometric devices, modified scan bodies, artificial landmarks);
- material and fabrication method (resin-based, metallic, 3D-printed, prefabricated);
- configuration (full-arch versus non-continuous or segmental connections); and
- reported effects on digital impression accuracy (trueness, precision, angular deviation, scanning time, and fit outcomes).

Results were summarized to highlight patterns, similarities, and divergences across the literature, with particular emphasis on identifying recurring design principles, scanner-dependent effects, and clinical or experimental conditions under which splinting or auxiliary devices were beneficial, neutral, or detrimental. No attempt was made to rank interventions or infer-superiority, as this review aimed to map the available evidence rather than to evaluate effectiveness.

No meta-analysis or formal risk-of-bias assessment was conducted, in accordance with established scoping review methodology and PRISMA-ScR recommendations.

## 3. Results

The initial electronic database search identified 2488 records, while no additional records were retrieved from grey literature or manual reference searching. After removing 722 duplicates, 1766 records were screened by title and abstract. Of these, 99 full-text articles were assessed for eligibility, and 26 were excluded for reasons such as absence of data on splinting implant scan bodies, and inadequate outcomes. A total of 73 studies met the inclusion criteria and were included in this scoping review. The study selection process followed the PRISMA-ScR framework and is summarized in the PRISMA flow diagram (Figure 1). Study characteristics and extracted data are presented in Table 1 and Table S1.

The vast majority of the included studies (n = 71) investigated fully edentulous arches, while only two evaluated partially edentulous configurations [59,75], and one study assessed implant analog assemblies without direct reference to edentulism [89]. Most studies were conducted in vitro (n = 60), with 12 clinical studies and 1 mixed-design investigation that included both laboratory and clinical components [68]. Among clinical studies, nine were non-randomized clinical trials, one was a case series, and two were retrospective evaluations.

Regarding arch distribution, 37 studies examined the maxilla, 26 the mandible, and six included both jaws [31,39,46,50,68,87]. Four studies did not explicitly specify the jaw or were not applicable due to their experimental setup [49,58,73,88].

Scan body materials varied across the included studies. PEEK scan bodies were used in 28 studies, reflecting their widespread adoption in digital implant workflows due to favorable optical and mechanical properties. Titanium scan bodies were reported in 14 studies [23,24,25,39,50,51,54,56,63,70,82,83,84,95], typically in modified or extended designs to improve geometric detectability and reduce stitching errors. The remaining studies used other materials or did not specify the scan body composition.

Across the included literature, splinting or auxiliary scanning aids were implemented with substantial methodological variability. Full-arch connection of intraoral scan bodies (ISBs) was employed in 38 studies, whereas 27 studies used non-continuous or segmental connections between ISBs. An additional five studies included both full-arch and non-continuous approaches as part of comparative experimental groups, and four were not applicable due to model design or lack of splinting components.

A broad spectrum of materials and fabrication methods was used to connect or augment scan bodies. Rigid frameworks or devices were reported in five studies, while seven used traditional floss-and-resin splinting. Three studies reported methods that did not apply to conventional material categorization. Notably, 34 studies employed 3D-printed splints or devices, and 33 studies used auxiliary scanning aids, such as geometric attachments, cross-arch bars, calibration jigs, or artificial landmarks. When categorized by resin involvement, 38 studies utilized resin-based materials (autopolymerizing, light-cured, or flowable resins) either as the primary splint or as a stabilizing medium.

Across nearly all studies, splinting was performed using one of three primary strategies:

1. Rigid or semi-rigid auxiliary geometric devices (AGDs) fabricated via 3D printing, milling, or modular resin assembly;
2. Direct splinting of standard scan bodies using floss, pattern resin, injectable or light-cured composites, or prefabricated bars; and
3. Modification of scan bodies themselves, including horizontal geometries, wings, CAD/CAM extensional structures, artificial landmarks, or geometric attachments that increased surface heterogeneity.

Across all included studies, splinting or the use of auxiliary scanning aids generally improved the accuracy and performance of complete-arch digital impressions. Specifically, 54 studies reported improved outcomes—including enhanced trueness, precision, reduced deviations, fewer rescans, shorter scanning time, or improved passive fit—when splinting or scanning aids were used. In 13 studies, no significant difference was detected between splinted and non-splinted conditions [31,32,38,42,44,49,50,53,59,63,65,68,80]. Conversely, eight studies reported a negative influence, where splinting or the chosen auxiliary device increased scanning deviations or interfered with scan-body detection [40,43,52,57,67,71,72,90].

The included studies collectively examined implants across mandibular and maxillary models, polyurethane, PMMA or gypsum casts, titanium master models, and clinical patient cases. The number of implants per arch ranged from two to eight, with most studies evaluating four- to six-implant configurations, including both parallel and tilted distal implants. A diversity of implant systems and scan body designs were represented.

## 4. Discussion

This scoping review mapped and synthesized the rapidly expanding literature on splinting materials, scan body modifications, and auxiliary devices designed to enhance the accuracy of complete-arch digital implant impressions. Across a large methodological spectrum—from benchtop models to in vivo clinical evaluations—the collective evidence demonstrates that the accuracy of intraoral scanning, even in challenging edentulous full-arch scenarios, remains highly dependent on the availability of stable geometric landmarks. Splinting strategies, particularly rigid cross-arch devices or geometrically enriched scan bodies, consistently improved stitching reliability and reduced cumulative deviations, especially over long scanning spans and in the presence of tilted distal implants. These findings reinforce the core biomechanical principle that improving the distribution of reference points mitigates the limitations inherent to current image-stitching IOS algorithms.

The present findings suggest that splinting or the use of auxiliary scanning aids enhanced digital accuracy in the majority of studies: 52 studies reported clear improvements [20,22,23,24,25,29,30,33,35,36,37,39,41,44,45,46,47,48,51,54,55,56,58,59,60,61,62,63,64,65,66,68,69,70,73,75,76,78,79,81,82,83,84,85,88,89,90,91,92,93,94,95], 6 found no significant differences [31,32,38,42,53,80], and 6 reported detrimental effects [40,43,52,57,71,72]. This distribution strongly suggests that splinting is generally advantageous, though not universally. These findings are consistent with recent evidence [96]. The variability in outcomes largely reflects differences in device rigidity and geometric design, as rigid, multi-planar, or well-engineered splints consistently enhance landmark availability for stitching. In contrast, flexible, unstable, or overly bulky devices may deform, interfere with scan-body recognition, or introduce new sources of error [24,57]. These findings align with broader evidence that cross-arch or geometrically enriched frameworks significantly reduce linear and angular deviations in full-arch implant scans, confirming the critical role of structural stability in digital accuracy [97,98]. Importantly, several studies reported contradictory outcomes for comparable splinting strategies depending on the intraoral scanner and experimental context [24,38]. Similar devices improved accuracy in some IOS systems while remaining neutral or even detrimental in others, highlighting pronounced scanner-specific interactions (e.g., Primescan, TRIOS, iTero, and Medit platforms) [24,38,43,47,51]. These findings emphasize that splinting benefits cannot be assumed a priori and must be interpreted in relation to both device design and IOS technology.

In addition, scan-body material and macro-geometry substantially influence how effectively scanners capture implant position [98,99]. Studies have reported that PEEK scan bodies often exhibit favorable optical properties [100], while titanium allows for more stable extensions or calibrated frameworks, though both materials can vary in performance depending on surface characteristics and dimensional design [101,102]. Research has also demonstrated that modified scan bodies—such as those with wings, horizontal connectors, or extensional structures—can markedly improve trueness and precision by increasing geometric distinctiveness and reducing stitching ambiguity [69]. Conversely, simpler or low-contrast geometries may limit the scanner’s ability to maintain accuracy across edentulous spans [40].

Scanner-dependent behavior contributes significantly to inter-study variability [103,104]. Different intraoral scanners rely on unique optical technologies, capture speeds, and stitching algorithms, meaning that a splinting method that performs well with one system may not translate to another [24,43]. Comparative studies consistently show differences in trueness and precision among IOS devices when used for complete-arch implant impressions, with some scanners exhibiting greater resilience to long-span stitching errors and others being more sensitive to splint design, operator movement, or scan strategy [24,43,49,55]. Collectively, these technological and procedural factors explain why splinting improves accuracy in most—but not all—investigations. Rapid hardware and software evolution of intraoral scanners represents an important contextual factor when interpreting these findings. Improvements in optical sensors, image acquisition speed, and stitching algorithms over successive scanner generations may partially explain inter-study variability and limit the generalizability of results obtained with earlier IOS systems to current clinical practice.

Across all studies, the influence of scanner type was a consistent determinant of accuracy. Primescan, iTero, and TRIOS scanners often yielded the highest trueness values, with Primescan frequently outperforming the others. Lower performance scanners exhibited greater sensitivity to splinting method and arch complexity. Several investigations reported high operator dependency for specific IOS–scan body combinations [24,43]. Differences in acquisition technology (video vs. confocal vs. LED triangulation), dynamic range, and image-processing algorithms may account for these variations.

Rigid auxiliary geometric devices were the most commonly investigated approach [29,30,80,87]. Notably, 33 studies used auxiliary devices, and 34 studies used 3D-printed splints or devices. The proliferation of printed, custom-fitted devices highlights a trend toward highly engineered, digitally planned splinting systems that integrate seamlessly into IOS acquisition workflows. These devices, ranging from denture-like frameworks to screw-retained cross-arch bars and geometric lattices, were consistently shown to enhance the trueness and precision of intraoral scans, particularly in long-span scans and for distal implants where stitching errors accumulate. Studies employing large, cross-arch devices reported substantial reductions in 3D deviation, improved inter-implant distance measurements, and marked decreases in angular error [37,54,94]. Designs that shortened the scanning path and provided additional reference points for the stitching algorithms were especially effective [60,69,89]. It has also been suggested that AGDs decreased rescanning frequency and reduced total scanning time, supporting their value both technically and clinically [29,31,39,53]. However, the performance of AGDs varied by scanner type, with some devices improving accuracy on specific scanners but not others [83]. One study noted that an AGD actually worsened angular deviation, highlighting that auxiliary structures are not universally beneficial and must be tailored to scanner characteristics [40]. However, these findings should be interpreted with caution, as the available evidence is predominantly derived from in vitro investigations. Laboratory models cannot fully replicate clinical conditions such as soft-tissue compressibility, saliva, patient movement, limited scanning access, or operator variability, all of which may influence intraoral scanner performance. Consequently, the apparent benefits observed for certain splinting strategies under controlled conditions may not translate directly to routine clinical practice.

Direct splinting using floss and resin, composite resin, or light-polymerizing materials showed mixed outcomes. Some studies confirmed that rigidly splinting adjacent scan bodies improved trueness, particularly when the splint was sectioned and reconnected to compensate for polymerization shrinkage [31,48,88]. Modular chain splints and prefabricated resin bars often produced deviations comparable to conventional splinted open-tray impressions [44,49]. Conversely, other research groups demonstrated that splinting ISBs with floss and resin materials frequently resulted in reduced accuracy [58,71]. Flexible structures introduced instability, deformation, or interference with the scan bodies themselves, leading to greater stitching distortion [57]. A possible explanation was that splinting materials impeded each IOS software’s ability to recognize ISBs, resulting in worse accuracy than non-splinted digital scans [57].

A different approach involved modifying the scan bodies to enhance their geometry. Horizontal scan bodies, metallic L-shaped attachments, PEEK extensions, winged designs, and CAD/CAM extensional structures consistently improved accuracy without requiring cross-arch splinting [24,83,84,86]. These extensions enriched geometric complexity in edentulous spans, improved image stitching, and reduced long-span linear and angular deviations. Several studies reported that modified scan bodies performed as well as or better than conventional splinted impressions, particularly for distal implants and angulated configurations [46,53,58]. Horizontal or palatally connected designs produced significant improvements in both trueness and precision across several scanner systems, while titanium extensional structures achieved accuracy close to that of conventional splinted methods [83,95].

Some studies introduced artificial landmarks placed not on the scan bodies but on the edentulous mucosa, such as resin spheres or pressure-indicating pastes [58,60,75]. These techniques aimed to compensate for the lack of anatomical reference points in fully edentulous arches. Their effects were highly scanner-dependent: in some protocols, artificial landmarks yielded accuracy comparable to splinting, while in others they offered no improvement or even degraded accuracy [38,91].

The impact of implant angulation, inter-implant distance, and number of implants was also extensively documented. Accuracy decreased as the measured distances increased, especially in cross-arch scans [49]. Splinting—whether via AGDs or modified scan bodies—consistently mitigated these distance-related inaccuracies [52]. Tilting distal implants exacerbated linear and angular deviations; however, rigid splinting devices significantly reduced these errors [37,81]. Increasing the number of implants tended to improve precision in some studies, as additional scan bodies provided more anchor points for stitching [67], although contradictory results were also reported [64].

When digital splinting was compared to conventional impressions, results varied depending on the specific protocol. Several studies found that conventional splinted open-tray impressions remained the most accurate method [31,33,78]. Others, especially those using rigid or calibrated digital frameworks, reported that digital methods were equal to or superior to the conventional approach, with improved passive fit confirmed clinically through Sheffield tests, radiographs, or torque assessments [27,65,68,69,70,95]. In vivo studies demonstrated high prosthetic success rates, including reports of 100% survival with digitally fabricated full-arch prostheses produced using calibrated splinting frameworks [65,84,89].

A key finding of this scoping review is the marked methodological heterogeneity among the included studies, which limits direct comparison between splinting strategies and precludes definitive conclusions regarding clinical superiority. Considerable variation existed in implant configurations, scan body and splinting designs, auxiliary device geometry, intraoral scanner technologies, and accuracy assessment methods, with outcomes reported using non-standardized metrics. This variability has direct clinical relevance, as digital implant impression accuracy is highly sensitive to cumulative stitching errors, scanner-specific algorithms, material optical properties, and geometric reference enrichment. Consequently, a strategy that improves trueness or precision with one scanner may be ineffective or detrimental with another, and flexible or optically interfering materials may compromise accuracy. Overall, no single splinting or scan-aid approach can currently be recommended as universally superior for complete-arch digital implant impressions, although rigid and geometrically enriched devices appear to perform more consistently, particularly in long-span or angulated configurations. The absence of standardized evaluation protocols remains a major barrier to translating these findings into clear clinical guidelines.

From a clinical perspective, the findings of this scoping review suggest that the use of splinting strategies or auxiliary geometric aids may be beneficial primarily in demanding scenarios, such as complete-arch or long-span implant rehabilitations, where cumulative stitching errors are most likely to occur. In routine short-span or partially edentulous cases, conventional intraoral scanning with standard scan bodies generally appears sufficient, and additional splinting may offer limited clinical advantage.

It is important to distinguish between evidence derived from in vitro investigations and that supported by clinical data. The majority of strategies demonstrating improved trueness or precision—particularly rigid auxiliary geometric devices, calibrated frameworks, and modified scan bodies—have been evaluated predominantly under controlled laboratory conditions. In contrast, clinical evidence remains limited but suggests that well-designed rigid or calibrated devices may improve scanning efficiency, passive fit, or both, whereas flexible or resin-based splints have shown inconsistent or unfavorable results in vivo.

For daily clinical practice, these findings indicate that dentists should adopt splinting or auxiliary scanning strategies selectively, considering case complexity, implant distribution, scanner system, and workflow familiarity. Until further high-quality clinical studies become available, rigid and geometrically enriched devices with existing clinical validation appear to be the most promising options for complete-arch digital implant impressions.

One of the strengths of this scoping review is that it provides a broad and current synthesis of splinting strategies and auxiliary devices aimed at improving intraoral scanning accuracy in full-arch implant rehabilitation. By including 73 studies across in vitro, clinical, and mixed designs, it captures approaches ranging from floss-and-resin splinting to 3D-printed calibration frameworks and modified titanium scan bodies. The structured extraction of splinting methods, connection patterns, scan-body designs, and IOS performance enabled a precise mapping of factors influencing accuracy. In addition, reporting quantitative descriptors strengthens interpretability and clinical relevance.

Several limitations should be considered when interpreting these findings. The predominance of in vitro studies limits direct extrapolation to clinical settings where saliva, movement, soft tissue, and access affect IOS performance, and the smaller number of clinical studies restricts generalizability. Marked heterogeneity in splinting materials, scanners, implant systems, and outcome metrics prevented quantitative analysis and limited direct comparison. Inconsistent reporting of scan-body material composition and geometry further complicates interpretation, and restricting inclusion to English-language publications may have led to the omission of relevant studies.

Future research should prioritize standardizing splint design parameters (rigidity, geometry, and material properties) and conducting scanner-specific compatibility studies to clarify when splinting is most beneficial. Larger clinical trials are needed to validate in vitro findings and evaluate passive fit, complications, efficiency, and patient-centered outcomes.

Splinting and AGD generally improve full-arch digital implant impression accuracy, especially in long spans and angulated configurations, with the most predictable gains observed for rigid, geometrically complex, or calibrated devices. Modified scan bodies may offer a less bulky alternative, while flexible or optically interfering materials can worsen deviations and should be avoided. Because outcomes are scanner-dependent, device selection and scanning strategy should be matched to the IOS system used. When appropriately implemented, digital splinting protocols can approach—and in some settings exceed—conventional splinted open-tray impressions while improving comfort and efficiency.

## 5. Conclusions

This scoping review mapped the existing evidence on splinting strategies, scan-body modifications, and auxiliary geometric devices used to improve the accuracy of complete-arch digital implant impressions. Most studies clustered around rigid or geometrically enriched approaches and reported improved accuracy, particularly in long spans and angulated configurations, whereas flexible splints showed inconsistent effects. However, the evidence is predominantly in vitro and strongly scanner-dependent, highlighting important gaps in clinical validation and the need for standardized evaluation protocols and further in vivo research.

## Figures and Tables

**Table 1:** Study characteristics and extracted data.

Author	Study Type	Sample/Jaw	No of Implants	Splinting Material/Scan Aid	Key Findings
Abdelrehim, et al., 2025 [29]	In vitro	3 mandibular casts	10 implants, Groups: 3	Custom rigid scan-aid apparatus (auxiliary geometric device, 3D printed)	Splinting with the apparatus significantly improved precision in all groups. Total scanning time was reduced for 4-implant groups. Time lost due to errors was reduced for all groups. Geometric heterogeneity and added reference points improved image stitching stability.
Abdelrehim, et al., 2025 [30]	In vitro	3 mandibular models	10 implants, Groups: (2IP, 4IP, 4IA)	Custom rigid auxiliary geometric device (3D printed)	Auxiliary geometric device improved 3D, linear, and angular accuracy. All non-splinted groups exceeded both linear and angular deviation limits. Posterior implants showed the highest errors in non-splinted scans
Ali, et al., 2024 [31]	Nonrandomized clinical trial	19 arches (4 Maxilla, 15 Mandible)	4 to 6 implants per arch	Floss and pattern resin	Conventional impressions were more accurate than splinted and unsplinted digital scans. No significant difference in positional or angular trueness between splinted and unsplinted. Splinting significantly reduced scan time. Maxillary scans faster than mandibular. Conventional impressions were more accurate than digital scans.
Almalki, et al., 2025 [32]	In vitro	1 maxillary typodont	6 implants (multiunit abutment analogs)	Dual-purpose scan jigs	No significant differences in accuracy between the two groups at any location. Both ISB types produced deviations within clinically acceptable ranges (<150 μm).
Anwar, et al., 2024 [22]	In vitro	1 maxillary model	4 implants (2 parallel anterior, 2 tilted posterior)	Specially designed geometric device (3D printed gray resin)	Both the geometric device and ISB modifications significantly improved trueness and precision compared to the control group. The geometric device had a greater positive impact on accuracy than the ISB modifications alone. The highest accuracy was achieved when using the geometric device and modified scan bodies simultaneously.
Arikan, et al., 2023 [33]	In vitro	1 maxillary epoxy resin model	4 implants (Canine and First Molar positions)	Custom 3D-printed auxiliary geometric appliance (AGA) fixed with light-polymerizing resin	Digital impressions without the auxiliary device had the worst accuracy. Use of the auxiliary geometric appliance significantly improved accuracy. Digital impressions were significantly less accurate than conventional splinted open-tray impressions or lab scan. All groups stayed within clinically acceptable misfit thresholds (<150 µm).
Asavanant, et al., 2025 [34]	In vitro	1 mandibular master cast	4 implants (Multiunit abutment analogs)	Group 2: Metal mesh and light-polymerizing acrylic resin (Bulk Fill Flowable Composite)/Group 3: Passively fitting interim prosthesis (Reverse scanbody protocol)	Conventional splinted open-tray: 0% misfit—best performance. Reverse scanbody: 20% misfit. OPTISPLINT splinted ISBs: 60% misfit. Conventional splinted open-tray impression + milled devices produced superior passive fit. Reverse ISB protocol outperformed splinted ISBs with auxiliary features.
Ashida, et al., 2025 [35]	In vivo clinical study	8 participants (Maxilla)	4 implants per patient	Custom assistive device: Milled Polymethyl methacrylate (PMMA) fixed with self-curing resin	Digital impressions with the assistive device showed significantly higher precision compared to the non-splinted digital group and the conventional verification cast method. The device’s design, which crossed the palate to connect posterior implants, significantly reduced the scanning path length and accumulated errors. Precision was in vivo lower than in vitro, but still improved with assistive device.
Ashraf, et al., 2023 [36]	In vitro	2 maxillary models	4 implants each	Custom modular chain device: 3D printed resin elements assembled to form a chain	Splinting ISBs with the modular chain device significantly improved trueness and precision compared to the non-splinted. Primescan showed the highest trueness and precision, followed by Trios 4. Medit i600 showed significantly lower accuracy. The distal implant angulation (30° vs. 45°) had no significant effect on trueness or precision.
Ashry, et al., 2025 [37]	In vitro	1 Maxillary PMMA model with soft tissue replica	4 implants	Custom PEEK scan body accessories (milled 8-mm arms friction-fitted to the scan bodies)	Accessories significantly improved the overall scanning precision. They also significantly reduced 3D deviation at the most distal ISB, where errors were highest, though the overall difference was not statistically significant. Significant improvement in linear trueness was found for specific inter-implant distances. No difference in angular deviation between groups.
Azevedo et al., 2025 [24]	In vitro	1 mandibular definitive cast	6 implants	Not applicable	All factors significantly affected trueness (operator, ISB, IOS). Horizontal ISBs were more accurate than the vertical ISB. Primescan delivered the best trueness. iTero Element 5D + vertical ISBs had worst accuracy and strong operator variability.
Azevedo, et al., 2024 [38]	In vitro	1 mandibular definitive cast	6 implants	Group splinted: Orthodontic wire and pattern resin/Artificial landmarks group: made of light-polymerized resin placed on mucosa	No statistically significant differences in trueness between the conventional, splinted and artificial landmark techniques. Scanner significantly influenced trueness. Primescan showed higher trueness than iTero 5D and TRIOS 4. While splinting generally did not improve trueness for most scanners, it improved trueness specifically for the TRIOS 4.
Campana, et al., 2024 [39]	Case Series clinical study	12 patients (6 Maxilla, 6 Mandible)	4 to 6 implants per arch	Universal Scan Template (UST^®^): Modular 3D printed Rigid Gray Resin stabilized with orthodontic elastics	The use of the UST resulted in 100% passive fit for the manufactured titanium bars clinically, compared to only 20% passive fit without UST. Scan time was significantly reduced with the use of UST. Significant distortion was measured between the STL files acquired with and without UST.
Canullo, et al., 2024 [40]	In vitro	1 maxillary model	4 implant replicas	Auxiliary Geometric Device (AGD): Milled Polymethylmethacrylate (PMMA) fixed with cyanoacrylate adhesive	AGD worsened angular deviation overall. AGD did not improve accuracy for TRIOS or CS. Full-arch scans can still be clinically acceptable without AGD.
Chen, et al., 2025 [41]	In vitro	1 maxillary model	6 implants	Calibration Device (Scan Aid—SA): 3D printed resin in Gray, Tan, or White. Fixed with occlusal registration polyvinyl siloxane (PVS).	The CISP method provided significantly improved trueness and precision over IOS alone or IOS with the scan aid only. CISP achieved superior accuracy at distal sites. At the most distal site, CISP trueness was significantly lower than IOS.
Cheng, et al., 2024 [42]	In vitro	Maxillary model made from an aerospace-grade aluminum block	6 implants	Commercially available splinting attached directly to the scan body’s cross-hole	Stereophotogrammetry (SPG) exhibited superior trueness and precision. The splinted ISBs showed no statistically significant difference in trueness or precision when compared to the non-splinted IOS or the conventional impression. Intraoral scanning displayed a tendency for angular and linear deviations in the cross-arch region.
Denneulin, et al., 2023 [43]	In vitro	1 mandibular model	Configurations tested: 6 implants, 4 implants (short arch), 4 implants (long arch).	Blue suture thread	Splinting with suture thread negatively affected accuracy with Trios 3 and had no significant effect with Primescan. Trueness and precision were significantly better with 6 implants compared to 4 implants for both scanners.
Eddin and Önöral, 2024 [44]	In vitro	1 mandibular cast	4 implants	PR: Dental floss and pattern resin/CR: Dental floss and composite resin/AA: 3D printed auxiliary apparatus (PLA)/CSB: Custom scan bodies with extensions (PLA).	The conventional splinted impression showed the lowest distortion values (best trueness) overall. The 3D printed auxiliary apparatus group had distortion levels comparable to the conventional method and significantly better than the non-splinted group. All splinting/modification methods significantly improved trueness compared to the non-splinted SB group.
Eid, et al., 2024 [45]	In vitro	1 maxillary model	4 implants	Group Aux: Custom geometric auxiliary device (3D printed resin) with tooth-like landmarks.	Both digital groups (with and without the device) resulted in a more accurate fit (smaller gaps) than the conventional impression group. The auxiliary device improved the passivity of the fit compared to the standard digital scan, though both were within clinically acceptable limits.
Eldabe, et al., 2025 [46]	In vivo prospective comparative study	7 arches (2 Maxillae, 5 Mandibles) in 6 patients	4 to 5 implants per arch (Total 34 implants)	A novel scan body modified with directly connected tooth-shaped objects acting as stable artificial landmarks to fill the gaps between scan bodies.	The Tooth-Modified Scan Body (TMSB) group showed significantly lower Euclidean deviation compared to the Conventional Scan Body (CSB) group. TMSB showed significantly lower angular deviation and reduced scanning time compared to CSB.
Farah, et al., 2025 [47]	In vitro	1 maxillary cast	4 implants	Custom 3D-printed Geometric Attachments (GAs) (acrylate-based resin)	The use of GAs significantly reduced deviations (improved accuracy) for both scanners. iTero exhibited significantly lower mean 3D surface and linear deviations (better accuracy) than Omnicam. ISB angulation (0° vs. 30°) did not significantly impact scan deviations.
Farahat and El Saaedi, 2025 [48]	In vitro	1 mandibular resin model	4 implants	Group III (Digital Splinted): Dental floss and injectable hard-liner resin material	Conventional impression showed the highest trueness. Splinting ISBs improved trueness compared to the non-splinted digital group.
Ferrini, et al., 2024 [49]	In vitro	1 titanium master model	6 implants	Dental floss and light-curing resin, sectioned and reconnected to reduce shrinkage.	Significant differences in trueness among the tested scanners, with TRIOS and Medit i700 wireless yielding the worst trueness values for long distances. No significant differences were found in precision. Increasing the measuring distance led to a decrease in both trueness and precision for all scanners.
Fu, et al., 2024 [50]	In vivo clinical study	15 patients, 22 arches (9 upper, 13 lower)	4 to 6 implants per arch (Total 115 implants)	IOS group: Prefabricated bar splints selected according to distance and screwed into the scan bodies.	IOS showed significantly greater angle deviation and RMS error than SPG. IOS with prefabricated aids was the most efficient workflow.
Fu, et al., 2025 [51]	In vitro	4 maxillary master models	4 and 6 implants	IOS-T/M/A with aid: Titanium-prefabricated bar splints (scan aids) screwed into the scan bodies	Scanning with prefabricated aids improved both trueness and precision for all three IOSs compared to without aids. With aids, IOS-A (Aoralscan 3) and IOS-M (Medit i700) showed better trueness than IOS-T (TRIOS 5). IOS-M achieved the most improvement with aids. A significant correlation was found between virtual RMS errors and physical framework misfit.
Garbacea, et al., 2021 [52]	In vitro	1 maxillary master cast	6 implants	Complete arch splint: 3D printed resin with minimal irregularities like natural teeth geometry landmarks added to the periphery.	Splinting ISBs did not improve scan accuracy. Trios and Atos scanners recorded lower deviations than True Definition (TD) and Dental Wings (DDW), especially in splinted groups.
García-Martínez, et al., 2022 [53]	In vitro	1 mandibular master model	6 implants	Customized over-scan body rings (COR): 3D printed (Dental Model resin) rings with irregular patterns snapped on ISBs	The COR system reduced the number of rescans required and total scanning time. No differences were found in terms of linear or angular trueness and precision. The use of COR improved efficiency without impacting accuracy.
Gianfreda, et al., 2025 [25]	In vitro	1 maxillary model (gypsum)	4 implants	Auxiliary Geometric Device (AGD): A screwable extension that creates an L-shaped scan body.	The AGD significantly enhanced trueness and precision of full-arch implant impressions when compared with conventional ISBs, reducing stitching errors and enhancing ISB stability during full-arch digital impressions.
Gómez-Polo, et al., 2024 [20]	In vitro	2 maxillary casts	8 implants (4 implants each)	Splinted Digital: Splinting framework attached to Ti abutments with autopolymerizing acrylic resin	Conventional impression showed the best trueness and precision. The splinted digital method had better trueness and precision than the non-splinted group, which had the worst accuracy. Parallel implants had better trueness and precision than nonparallel implants (up to 30°).
Huang et al., 2020 [54]	In vitro	1 mandibular acrylic model with soft tissue replica	4 implants	Custom CAD/CAM titanium alloy scan bodies	Conventional splinted open-tray impressions were more accurate than all digital impression groups. The design of the extensional structure significantly improved precision compared to original and non-extended ISBs. Trueness of the extended ISBs was comparable to the conventional impression and better than the other digital groups.
Ileri, et al., 2024 [55]	In vitro	2 maxillary model	8 implants (4 implants each)	Group 1: Artificial acrylic tooth used as a reference landmark. Group 2: Custom 3D printed Thermoplastic Polyurethane auxiliary geometric device.	The use of the custom auxiliary device improved trueness for all scanners compared to standard scanning, even in angulated cases. Primescan showed the highest trueness overall. Trios 3 showed the most significant improvement with the AGD, reducing deviations from unacceptable to acceptable levels. Primescan was the fastest scanner overall.
Iturrate, et al., 2019 [56]	In vitro	1 stainless-steel maxilla model	4 machined cylinders simulating scan bodies	Custom Auxiliary Geometric Device (AGD): A 3D-printed device shaped like a conventional complete-arch dentureand fixed with light-polymerizing resin	AGD improved trueness in all reference distances compared to scanning without it. The AGD improved precision in 4 out of 5 reference distances. Accuracy decreased as the length of the measured distance increased for both groups, but the AGD group maintained better accuracy over longer spans.
Iturrate, et al., 2019 [23]	In vitro	1 stainless-steel maxilla model	4 machined cylinders simulating scan bodies	Auxiliary Geometric Device (AGD): A custom 3D-printed device made of Acrylonitrile Butadiene Styrene simulating jaw with teeth.	The AGD significantly improved trueness for all reference distances. Precision was significantly improved with the AGD. Accuracy worsened as the scanning distance increased, but the AGD mitigated this effect.
Kalayci, et al., 2025 [57]	In vitro	1 mandibular master model	4 implants	Group C (Two-stage with markers): Buccal rubber reference markers placed between scan bodies during the second stage of scanning. Flexible auxiliary marker	Single-stage scanning (Group A) yielded the highest accuracy (lowest linear deviation) compared to two-stage methods. The addition of flexible rubber markers (Group C) significantly decreased accuracy. The flexible nature and lack of structural integrity of the markers were cited as reasons for the failure to improve stitching.
Kanjanasavitree, et al., 2022 [58]	In vitro	1 edentulous model	4 implants	FL Group: Dental floss tied between scan bodies and fixed with pattern resin. LD Group: Liquid Dam markers placed on the edentulous ridge (not a splint). PIP Group: Pressure-Indicating Paste brushed over the ridge (not a splint).	The markers group with the Quadrant scanning pattern showed the highest accuracy. The Floss/Resin splinting group did not improve accuracy and, in some combinations showed lower trueness compared to the LD group.
Kao, et al., 2023 [59]	In vitro	1 maxillary stone model	4 implants	An elastic orthodontic power chain stretched between scan bodies and covered with flowable resin	Splinting did not significantly improve accuracy for the short span. Splinting significantly improved: Angular trueness and linear precision for 4-unit span. The benefit becomes significant at >22.93 mm inter-implant distance.
Ke, et al., 2023 [60]	In vitro	1 mandibular stone cast	4 implants	Prefabricated landmarks: Custom 3D printed resin (collar + plate with letter patterns) attached to scan bodies with resin	Digital scans were more accurate than the splinted open-tray impression method. Prefabricated landmarks significantly improved the trueness and precision of full-arch scans. Landmarks enriched curvature variations in edentulous areas, improving image stitching
Kernen, et al., 2022 [61]	In vitro	1 maxillary gypsum cast	6 implants	Universal scan aid: Custom 3D printed device (brace + connector + bridge) Tested 3 designs (circular, square, irregular) and 3 resin colors (beige, gray, white)	The use of the universal scan aid improved trueness. The irregular design in beige color showed the highest trueness, but the material was brittle (high fracture rate). Precision was generally lower (worse) or equal when using the scan aid compared to unsplinted scans. The gray irregular scan aid was concluded to be the best overall option due to good trueness and superior fracture resistance (clinical applicability).
Lam, et al., 2025 [62]	In vitro	1 mandibular stone cast	4 implants	Auxiliary Geometry Part (AGP): Custom 3D printed denture-like framework	AGP showed significantly superior precision (for distance measurements compared to both non-splinted IOS and conventional impressions. The AGP acted as a reference structure, reducing misalignment and enhancing scanner precision for full-arch cases
Laureti, et al., 2025 [63]	In vitro	1 mandibular definitive cast	4 implants	Not applicable	Horizontal ISBs did not perform better than vertical ones in all scenarios. Significant interactions among ISB design, scanner type, and operator affected trueness. All discrepancies were within clinically acceptable limits (<150 µm).
Li, et al., 2024 [64]	In vitro	1 standard maxillary cast (printed resin)	Tested 4 and 6 implants (Subgroups: 6 implants, 4 implants short span, 4 implants long span)	Modified ISBs—Titanium alloy with anodized coatings and wing-like extensions	Modified ISBs showed significantly better trueness and precision than conventional ISBs. The accuracy of the modified scan bodies was not significantly affected by the number of implants (4 vs. 6) or the scan distance.
Li, et al., 2024 [65]	In vitro	1 maxillary model	6 implants	Group IOS-SA/CISP: Custom 3D-printed Calibration Jig (Model resin) with unique 3D pattern, secured around ISBs with polyvinylsiloxane	The Calibrated Intraoral Scan Protocol (CISP) showed comparable overall trueness and precision to the Conventional group. CISP significantly outperformed both IOS and IOS-SA in the Overall Fit Test. CISP achieved superior precision compared to all other groups in the Virtual Sheffield Fit Test.
Liu, et al., 2025 [66]	In vitro	3 maxillary models	14 implants (4, 4 and 6 implants)	Customized 3D printed auxiliary devices (photopolymerizable resin). Tested various designs, notably cube-like and hemisphere-like markers	The use of auxiliary devices significantly enhanced the trueness and precision. The cubic and hemisphere marker designs were the most effective, particularly over the longest spans. The 2.5 mm wide devices significantly outperformed the 5 mm wide devices.
Liu, et al., 2024 [67]	In vitro	3 3D-printed maxillary casts	14 implants	Prefabricated aid: Prefabricated bar	IOS (with prefabricated aids) had the lowest accuracy. Tilted implants significantly increased distance deviation compared to parallel implants. An increased number of implants was associated with improved precision.
Lu, et al., 2025 [68]	In vitro and In vivo	In vitro: 1 edentulous maxillary model./In vivo: 12 patients (maxilla/mandible)	4 implants (in vitro) 4 or more implants (in vivo)	DMC = cylindrical + metal crossbar DW = cylindrical + digital wings crossbar DRC = cylindrical + 3D-CRC (custom resin crossbar)	The use of DRC mitigated the negative impact of factors like implant number/parallelism on accuracy.
Lyu, et al., 2025 [69]	In vitro	1 acrylic resin mandibular model	4, 5, and 6 implants (tested in varying spans)	Consumable auxiliary device: O-I buckle (3D printed Model Resin/acrylates). Consists of a clamp (“O”) and a connecting rod (“I”) with an uneven surface	The O-I buckle significantly improved the trueness and precision of intraoral scanning compared to non-aided IOS, especially for long spans. For 4 and 5 implants, the accuracy of IOS with the O-I buckle was comparable to the Conventional Impression (CI). For 6 implants, IOS with the O-I buckle was slightly less accurate than CI.
Masu, et al., 2021 [70]	In vitro	1 maxillary model	4 implants	Custom-designed milled PMMA assistive devices (fixed with self-curing acrylic resin)/Type 1: bar connecting neighboring ISBs only/Type 2: Same neighboring connections as Type 1/PLUS: Posterior crossbar. Two perpendicular branches extending from this crossbar toward anterior implants	The Type 2 assistive device significantly improved impression precision compared to Type 0 (no aid). The Type 2 cross-arch design, which added a shorter scanning path over the palate, was the most effective configuration.
Mizumoto, et al., 2020 [71]	In vitro	5 polyurethane maxillary models	20 implants (4 implants each)	GB: Glass fiduciary markers on ridge/PP: Pressure-indicating paste on ridge/palate/FL: Floss tied between ISBs	The ZI ISB had significantly less distance deviation than AF. Splinting with floss resulted in significantly more distance deviation. Surface modifications did not improve accuracy. ISB type significantly affected scan time.
Nedelcu, et al., 2023 [72]	In vivo clinical study	5 participants (Maxilla)	30 implants (6 implants each)	Group DF: Dental floss tied around ISBs. Group SP: Bis-acrylic composite splint (Protemp 4) applied around ISBs	The non-splinted group showed the best trueness and precision. Splinting with floss (DF) or acrylic (SP) did not improve accuracy; the SP group had the worst trueness.
Nulty, et al., 2024 [73]	In vivo retrospective pilot study	10 patients (arch not specified)	24 implants	Scan Ladder (novel device): 1. Indirect variant (attached to conventional ISB). 2. Direct variant (titanium reusable matte surface ISB)	The Scan Ladder significantly improved the accuracy of Primescan and Medit i900 compared to scanning without aids. Traditional scanning without aids showed significantly higher deviations. The Elite IPG scanner demonstrated the highest trueness.
Önöral and Çakır, 2024 [74]	In vitro	1 mandibular master cast	5 implants	Custom 3D printed scan aids (PLA plus) in 5 different colors: Beige, Grey, White, Red, Blue.	Color significantly influenced trueness. The Grey scan aid group outperformed all other colors, exhibiting the lowest Angular and Linear deviations. Blue scan aids showed the highest distortion/lowest accuracy. Beige and White aids were better than Red and Blue but generally worse than Grey.
Önöral, et al., 2025 [75]	In vitro	1 maxillary master cast	2 implants	Prefabricated Auxiliary Device (PAD): 3D printed PLA+ scan aids (indented and plain versions) fitting on ISBs	All linear Deviations were clinically acceptable (<100 μm), but angular deviations for No-PAD and Plain-PAD groups exceeded 0.5 degrees in some locations (clinically unacceptable).
Pan, et al., 2021 [76]	In vitro	1 maxillary model	6 implants	Custom 3D printed auxiliary devices (Resin): Group 1 (Base only), Group 2 (Base + Spheres), Group 3 (Base + Artificial Teeth).	All three auxiliary device designs significantly improved the accuracy of complete-arch scanning compared to the control. Group 1 (Base only) showed the best linear trueness in a single quadrant. Group 3 (Base + Teeth) demonstrated the best accuracy for cross-arch scanning (second quadrant). Group 2 (Spheres) showed the least angular precision.
Pereira, et al., 2022 [77]	In vivo clinical study	17 participants (Mandible)	3 or 4 implants each	Device: Custom device consisting of ball attachment, fixation support, and cylindrical connection	Digital impressions required significantly less time than conventional. Arches with 3 implants required shorter scanning time than with 4 implants. Patients reported higher satisfaction, comfort, and less pain with digital impressions.
Pereira, et al., 2022 [78]	In vivo clinical study	10 participants (Mandible)	40 implants (4 implants each)	Custom scanning device consisting of ball-shaped attachment + fixation support + cylindrical connection	The group using the scanning device showed significantly better trueness for linear displacements. The device effectively improved the trueness of capturing implant positions in edentulous arches.
Pereira, et al., 2022 [79]	In vitro	1 mandibular master model	4 implants	Device: Device consisted of ball-shaped fixation, fixation support, and cylindrical union bar.	3D Deviations: No statistically significant difference was found between intraoral and extraoral scanning for either method. Distance Measurements: Intraoral scanning with the device showed better trueness for capturing inter-implant distances compared to scanning with ISBs only. Scanning with the device accurately captured all tested inter-implant distances.
Pol, et al., 2024 [80]	In vitro	1 mandibular Resin model	4 implants	3D printed Modular Chain splint (Rigid splint designed in CAD, printed in resin)	No significant difference in accuracy between intraoral digital impression and traditional impression, with or without the use of the 3D printed modular splint. The use of the 3D printed modular splint might enhance accuracy compared to non-splinted methods.
Pozzi, et al., 2022 [81]	In vitro	1 mandibular PMMA model	4 implants	ISB Splinting (ISS): 3D printed modular chain secured to ISBs with light-curable flow composite	ISS significantly improved the overall accuracy of the complete-arch digital impression. The splinting technique mitigated deviations caused by implant angulation, depth, and inter-implant distance.
Retana, et al., 2023 [82]	In vitro	1 mandibular polyurethane cast	4 implants	Splinting bars: Custom 3D printed resin bars (square cross-section, random textures) secured to ISBs with clear polyvinyl siloxane material	Splinting ISBs significantly improved the trueness of complete-arch digital scans for all tested IOSs. Increasing the inter-implant distance significantly decreased the trueness of the digital scans. Splinting helped compensate for the lack of anatomical landmarks in the edentulous jaw by providing a better scanning route.
Revilla-León, et al., 2025 [83]	In vitro	1 maxillary stone cast	6 implants	Horizontal ISBs	The Apollo group (Horizontal ISBs) obtained significantly better angular trueness and precision compared to the Standard ISB group. The scanning technique (Standard vs. Horizontal ISB) and choice of IOS impacted the accuracy of complete arch implant scans
Revilla-León, et al., 2025 [84]	In vitro	1 maxillary stone cast	6 implants	Horizontal ISBs	The noncalibrated splinting technique had significantly better linear trueness than the non-splinting technique. iTero demonstrated the worst linear trueness, while TRIOS 5 had the worst linear precision. The novel horizontal ISB design (connecting in the center of the palate) provided clinically acceptable accuracy.
Revilla-León, et al., 2025 [85]	In vitro	1 maxillary stone cast	6 implants	Splinted ISBs (SSB): 3D printed framework connected with Pattern Resin/Calibrated Framework (CF): Calibrated metal framework connected with Pattern Resin.	SPG and the CF technique obtained the best linear and angular trueness and precision. The CF technique was 50% more accurate than the non-calibrated polymeric splinting framework. The non-connected ISB technique demonstrated the lowest linear trueness values.
Revilla-León, et al., 2025 [86]	In vivo clinical study	1 patient (Mandibular arch)	4 implants	IOConnect: Noncalibrated horizontal splinting ISB	All tested techniques recorded implant positions within clinically acceptable discrepancies.
Roig, et al., 2022 [27]	In vivo clinical study	12 participants (maxillary arch)	78 implants (5 to 6 implants each)	Auxiliary device: a prefabricated device with anatomic forms, luted to interim copings	The digitally processed prosthesis (using the auxiliary device) was preferred over the conventionally processed one. Its clinical fit was better than those from the conventional workflow. Sheffield test results were significantly better for the digital framework.
Rustichini, et al., 2025 [87]	In vivo Retrospective clinical study	37 patients (44 arches: 25 maxillae, 19 mandibles)	198 implants (4 to 6 implants each arch)	Calibrated Splinting Framework (CSF): A rigid, dimensionally stable framework used to splint scan bodies.	The full-digital workflow using the CSF resulted in a 100% prosthesis survival rate after 1 year. Optimal passive fit was achieved in 97.7% of cases. The CSF technique proved to be a clinically reliable and precise method for fabricating implant-supported full-arch fixed dental prostheses.
Rutkunas, et al., 2022 [88]	In vitro	10 pairs of implant analogs fixed to stainless steel bars	20 implants (2 implants per bar)	Splinting strategies: PLA: Impression plaster. PTR: Autopolymerizing acrylic resin, cut and rejoined. ILN: Light-cured tray material ILC: Light-cured tray material, cut and rejoined. SBR: VPS bite registration LXB: Bis-acryl bite registration PTP: Bis-acryl composite 3DP: 3D printed bar attached with autopolymerizing acrylic resin.	Splinting techniques with rigid materials, proper polymerization, and compensating for shrinkage (cut and rejoin) produced the best results. 3D printed splints showed good stability over time.
Tallarico, et al., 2020 [89]	In vitro	2 mandibular titanium models	10 implants (4 and 6)	Custom prosthetic-based impression template (3D printed)	The prosthetic template significantly improved trueness and precision for both 4 and 6 implant scenarios. The control group had significantly higher angular deviations and stitching errors (13 retakes required in control vs. 0 in test).
Wu, et al., 2024 [90]	In vitro	1 maxillary master model	4 implants	Prefabricated auxiliary devices (PADs) 3D-printed in different resins (Grey, Translucent, White, Yellow) and shapes (Cuboid, Cylinder, Sphere)	PADs significantly influenced accuracy based on optical property and shape. Translucent PADs performed worse (more deviations). Cylinder shapes showed lower angular trueness on the YZ plane. Optimal trueness found with White-Cuboid, Grey-Sphere, and Yellow-Sphere combinations.
Wu, et al., 2024 [91]	In vitro	1 maxillary model	4 implants	Prefabricated auxiliary devices (PADs) 3D-printed in resin/Group II: With artificial landmarks at the anterior region provided by 2 short PAD/Group III: With artificial landmarks at the posterior region provided by 2 long PAD/Group IV: With artificial landmarks at both anterior and posterior regions provided by 2 short and 2 long PAD	Group IV (landmarks at both anterior and posterior) achieved the highest accuracy and was comparable to conventional open-tray impressions (Group V). Posterior landmarks (Group III) were found to be more pivotal for accuracy than anterior ones. All PAD groups significantly outperformed the control.
Wu, et al., 2024 [92]	In vitro	1 maxillary model	4 implants	Prefabricated auxiliary devices (PAD) 3D-printed in resin; designs included long and short variations with cuboid extensions	The test group (with PAD) demonstrated significantly higher linear trueness and precision than the control group across all scanning patterns.
Wu, et al., 2023 [93]	In vitro	1 maxillary model	4 implants	Scan Body Clasp (SBC): A novel prefabricated auxiliary device 3D-printed in resin. Tested in Flat vs. Curved morphologies and Low (near mucosa) vs. High levels 5 study groups: CO (Control)—No SBC LC (Low Curved) HC (High Curved) LF (Low Flat) HF (High Flat)	Attaching SBCs significantly improved accuracy. SBCs located near the mucosa (Low level) resulted in superior trueness. Flat morphology resulted in better precision. The Low-Flat (LF) group achieved the best overall accuracy
Kurtulmus-Yilmaz, et al., 2025 [94]	In vitro	3 mandibular models (acrylic resin)	12 implants (4 implants each)	Custom scan aids fabricated via Fused Deposition Modelling (FDM) using a 3D printer	Scan aids significantly improved trueness in most sites, particularly for longer inter-implant distances. Without scan aids, deviations increased as inter-implant distance increased. AD values without scan aids were often above the clinical threshold, whereas deviations with scan aids were clinically acceptable.
Zhang, et al., 2024 [95]	In vitro	1 mandibular stone model	4 implants	Custom-designed ISBs with integral extensions: Group CSS (Straight extension) and Group CSA (Arcuate extension simulating arch shape)	The arcuate extension (CSA) significantly improved precision compared to original, straight extension, and no extension. CSA precision was comparable to conventional impressions (CIs).

## Data Availability

No new data were created or analyzed in this study. Data sharing is not applicable to this article.

## Supplementary Materials

The following supporting information can be downloaded at: https://www.mdpi.com/article/10.3390/ma19040664/s1, Table S1: Supplementary data extracted from the included studies.

## Abbreviations

The following abbreviations are used in this manuscript:

ISB	Intraoral Scan Body

## Appendix A. Detailed Search Strategies

The detailed database-specific strategies were as follows:

- PubMed (MEDLINE):
  Population: (((((((((dental implant[MeSH Terms]) OR (dental implant*)) OR (impression technique*)) OR (edentulous)) OR (edentulism)) OR (edentulous jaw[MeSH Terms]))
  OR (dental prosthesis, implant supported[MeSH Terms])) OR (dental prostheses,
  implant supported[MeSH Terms])) OR (implant supported restoration*)) OR (fixed
  implant denture*)
  Concept: (((((((((digital scan*) OR (intraoral scan*)) OR (optical impression*)) OR
  (digital impression*)) OR (dental impression technique[MeSH Terms])) OR (dental
  impression technique*)) OR (dental impression material[MeSH Terms])) OR (dental
  impression materials[MeSH Terms])) OR (dental impression*)) OR (implant impression*)
  AND
  (((((((((((((((((scanbod*) OR (implant scan body)) OR (implant scan bodies)) OR
  (intraoral scanbod*)) OR (scan abutment*)) OR (scan post*)) OR (scan flag*)) OR
  (scanning device*)) OR (auxiliary device*)) OR (scan aid)) OR (scan aids)) OR
  (prefabricated aid)) OR (splinting)) OR (splinting technique*)) OR (splinting material*))
  OR (digital impression coping*)) OR (splint* scanbod*)) NOT (pet scan)
  Context: (((((digital workflow) OR (digital dentistry)) OR (accuracy)) OR (precision)) OR
  (trueness)) OR (implant position)
- Embase:
  (‘dental implant’/exp OR ‘dental implant*’:ti,ab,kw OR ‘impression technique*’:ti,ab,kw OR edentulous:ti,ab,kw OR edentulism:ti,ab,kw OR ‘edentulous jaw’/exp OR ‘implant supported dental prosthesis’/exp OR ‘implant supported restoration*’:ti,ab,kw OR ‘fixed implant denture*’:ti,ab,kw) AND (‘digital scan*’:ti,ab,kw OR ‘intraoral scan*’:ti,ab,kw OR ‘optical impression*’:ti,ab,kw OR ‘digital impression*’:ti,ab,kw OR ‘dental impression’/exp OR ‘dental impression technique*’:ti,ab,kw OR ‘dental impression material’/exp OR ‘dental impression material*’:ti,ab,kw OR ‘implant impression*’:ti,ab,kw) AND (scanbod*:ti,ab,kw OR ‘implant scan body’:ti,ab,kw OR ‘implant scan bodies’:ti,ab,kw OR ‘intraoral scanbod*’:ti,ab,kw OR ‘scan abutment*’:ti,ab,kw OR ‘scan post*’:ti,ab,kw OR ‘scan flag*’:ti,ab,kw OR ‘scanning device*’:ti,ab,kw OR ‘auxiliary device*’:ti,ab,kw OR ‘scan aid’:ti,ab,kw OR ‘scan aids’:ti,ab,kw OR ‘prefabricated aid’:ti,ab,kw OR splinting:ti,ab,kw OR ‘splinting technique*’:ti,ab,kw OR ‘splinting material*’:ti,ab,kw OR ‘digital impression coping*’:ti,ab,kw OR ‘splint* scanbod*’:ti,ab,kw) NOT (‘positron emission tomography’/exp OR ‘pet scan’:ti,ab,kw) AND (‘digital workflow’:ti,ab,kw OR ‘digital dentistry’:ti,ab,kw OR accuracy:ti,ab,kw OR precision:ti,ab,kw OR trueness:ti,ab,kw OR ‘implant position’:ti,ab,kw)
- Scopus:
  (dental implant OR implant dentistry OR dental prosthesis OR edentulous)
  AND
  (digital impression OR intraoral scan OR optical impression OR digital scan)
  AND
  (scan body OR scan bodies OR scan abutment OR scan post OR splinting OR
  scanning device)
- Web of Science:
  TS = (dental implant OR implant dentistry OR dental prosthesis OR edentulous)
  AND TS = (digital impression OR intraoral scan OR optical impression OR digital scan)
  AND TS = (scan body OR scan bodies OR scan abutment OR scan post OR splinting)
  AND TS = (accuracy OR precision OR trueness)
