# Supplementary material for: Materials and Techniques for Splinting Scan Bodies: A Scoping Review
Source: Materials (Basel). 2026 Feb 9;19(4):664. doi: 10.3390/ma19040664 (PMC12942197; doi:10.3390/ma19040664)
Supplement: Supplementary file 1 [file materials-19-00664-s001.zip › materials-4093044-supplementary.pdf]

Preferred Reporting Items for Systematic reviews and Meta-Analyses extension for Scoping Reviews (PRISMA-ScR) Checklist

SECTION	ITEM	PRISMA-ScR CHECKLIST ITEM	REPORTED ON PAGE #
**TITLE**			
Title	1	Identify the report as a scoping review.	Page 1
**ABSTRACT**			
Structured summary	2	Provide a structured summary that includes (as applicable): background, objectives, eligibility criteria, sources of evidence, charting methods, results, and conclusions that relate to the review questions and objectives.	Page 1
**INTRODUCTION**			
Rationale	3	Describe the rationale for the review in the context of what is already known. Explain why the review questions/objectives lend themselves to a scoping review approach.	Page 2
Objectives	4	Provide an explicit statement of the questions and objectives being addressed with reference to their key elements (e.g., population or participants, concepts, and context) or other relevant key elements used to conceptualize the review questions and/or objectives.	Page 2
**METHODS**			
Protocol and registration	5	Indicate whether a review protocol exists; state if and where it can be accessed (e.g., a Web address); and if available, provide registration information, including the registration number.	Page 2
Eligibility criteria	6	Specify characteristics of the sources of evidence used as eligibility criteria (e.g., years considered, language, and publication status), and provide a rationale.	Page 3
Information sources*	7	Describe all information sources in the search (e.g., databases with dates of coverage and contact with authors to identify additional sources), as well as the date the most recent search was executed.	Page 3-4
Search	8	Present the full electronic search strategy for at least 1 database, including any limits used, such that it could be repeated.	Supplementary material
Selection of sources of evidence†	9	State the process for selecting sources of evidence (i.e., screening and eligibility) included in the scoping review.	Page 4
Data charting process‡	10	Describe the methods of charting data from the included sources of evidence (e.g., calibrated forms or forms that have been tested by the team before their use, and whether data charting was done independently or in duplicate) and any processes for obtaining and confirming data from investigators.	Page 4-5
Data items	11	List and define all variables for which data were sought and any assumptions and simplifications made.	Page 4-5
Critical appraisal of individual	12	If done, provide a rationale for conducting a critical appraisal of included sources of evidence; describe	NA

SECTION	ITEM	PRISMA-ScR CHECKLIST ITEM	REPORTED ON PAGE #
sources of evidence§		the methods used and how this information was used in any data synthesis (if appropriate).	
Synthesis of results	13	Describe the methods of handling and summarizing the data that were charted.	Page 4-5
**RESULTS**			
Selection of sources of evidence	14	Give numbers of sources of evidence screened, assessed for eligibility, and included in the review, with reasons for exclusions at each stage, ideally using a flow diagram.	Page 5
Characteristics of sources of evidence	15	For each source of evidence, present characteristics for which data were charted and provide the citations.	Page 5
Critical appraisal within sources of evidence	16	If done, present data on critical appraisal of included sources of evidence (see item 12).	NA
Results of individual sources of evidence	17	For each included source of evidence, present the relevant data that were charted that relate to the review questions and objectives.	Page 5-6
Synthesis of results	18	Summarize and/or present the charting results as they relate to the review questions and objectives.	Page 5-6
**DISCUSSION**			
Summary of evidence	19	Summarize the main results (including an overview of concepts, themes, and types of evidence available), link to the review questions and objectives, and consider the relevance to key groups.	Page 6-9
Limitations	20	Discuss the limitations of the scoping review process.	Page 9
Conclusions	21	Provide a general interpretation of the results with respect to the review questions and objectives, as well as potential implications and/or next steps.	Page 9
**FUNDING**			
Funding	22	Describe sources of funding for the included sources of evidence, as well as sources of funding for the scoping review. Describe the role of the funders of the scoping review.	NA

JBI = Joanna Briggs Institute; PRISMA-ScR = Preferred Reporting Items for Systematic reviews and Meta-Analyses extension for Scoping Reviews.
* Where *sources of evidence* (see second footnote) are compiled from, such as bibliographic databases, social media platforms, and Web sites.
† A more inclusive/heterogeneous term used to account for the different types of evidence or data sources (e.g., quantitative and/or qualitative research, expert opinion, and policy documents) that may be eligible in a scoping review as opposed to only studies. This is not to be confused with *information sources* (see first footnote).
‡ The frameworks by Arksey and O'Malley (6) and Levac and colleagues (7) and the JBI guidance (4, 5) refer to the process of data extraction in a scoping review as data charting.
§ The process of systematically examining research evidence to assess its validity, results, and relevance before using it to inform a decision. This term is used for items 12 and 19 instead of "risk of bias" (which is more applicable to systematic reviews of interventions) to include and acknowledge the various sources of evidence that may be used in a scoping review (e.g., quantitative and/or qualitative research, expert opinion, and policy document).

From: Tricco AC, Lillie E, Zarin W, O'Brien KK, Colquhoun H, Levac D, et al. PRISMA Extension for Scoping Reviews (PRISMAScR): Checklist and Explanation. Ann Intern Med. 2018;169:467–473. doi: 10.7326/M18-0850.

Table S1. Study characteristics and extracted data.

Author	Study type	Type of edentulism	Scan body type	Splinting length	Scanner	Control group
Abdelrehim et al., 2025 [29]	In vitro	Full	Scan Abutment (Megagen Implant Co Ltd.)	Full arch connection (apparatus seated onto all ISBs)	Medit i600 (Medit Corp)	Non-splinted scan bodies (without scan-aid apparatus)
Abdelrehim et al., 2025 [30]	In vitro	Full	Scan Abutment (Megagen Implant Co Ltd.)	Full arch connection	Medit i600 (Medit Corp)	Non-splinted scan bodies (without auxiliary geometric device)
Ali et al., 2024 [31]	Nonrandomized clinical trial	Full	CARES (Straumann Inc) and Elos (Nobel Inc)	Full arch connection (sectioned and reconnected)	TRIOS 3 (3Shape A/S)	Desktop scan (3Shape D900L) of a verified definitive stone cast with scan bodies
Almalki et al., 2025 [32]	In vitro	Full	Group 1: Standard Scan Bodies (SBs) (Neodent) Group 2: Dual-Purpose Scan Jigs (DPSJs) (TRUSS, DW Digital Solutions LLC)	N/A	CEREC Primescan (Dentsply Sirona)	Reference model scanned with a high-precision laboratory scanner (inEos X5).
Anwar et al., 2024 [22]	In vitro	Full	CopaSky (bredent medical) / Tested both standard and modified (with subtractive round depressions)	Full arch connection	CS3700 (Carestream Dental)	Non-splinted standard scan bodies (No Device - No Modification group)
Arikan et al., 2023 [33]	In vitro	Full	Medentika scan bodies (Medentika GmbH)	Full arch connection	Medit i500 (Medit Corp.)	Master model scanned directly with a laboratory scanner (inEos X5).
Asavanant et al., 2025 [34]	In vitro	Full	Group 2: OPTISPLINT (Digital Arches)<br><br>Group 3: RevEX Reverse Scanbodies (Straumann AG)	Full arch connection	TRIOS 4 (3Shape A/S)	Group 1: Conventional splinted open-tray impression
Ashida et al., 2025 [35]	In vivo clinical study	Full	Nobel Biocare scan bodies	Full arch connection (cross-arch design connecting adjacent scan bodies and crossing the palate between #15 and #25)	TRIOS 4 (3Shape)	1. Digital impression without assistive device (AD-)<br>2. Conventional impression using an implant indexing device (Verification Cast - VC), scanned with a coordinate measuring machine.
Ashraf et al., 2023 [36]	In vitro	Full	Cares NC mono-scanbody (Straumann)	Full arch connection	Primescan (Dentsply Sirona)<br><br>Trios 4 (3Shape)<br><br>Medit i600 (Medit Corp)	Non-splinted scan bodies
Ashry et al., 2025 [37]	In vitro	Full	CARES RC Mono Scan body (PEEK) (Straumann)	N/A (Individual extensions to reduce inter-implant	Medit i700 wireless (Medit Corp)	Group A: Standard scan bodies without accessories

				edentulous space)		
Azevedo et al., 2025 [24]	In vitro	Full	Vertical (V-ISB): Elos Accurate (V-EA)<br><br>Horizontal (Multi-unit Polo (H-NB) Nexus Scan Gauges (H-NS)<br>M6 Dental Multi-unit abutment (H-M6)<br>SmartFlags (H-SF)	N/A	iTero Element 5D (Align Technology)<br><br>Primescan (Dentsply Sirona)<br><br>TRIOS 3 (3Shape)<br><br>TRIOS 4 (3Shape)	Vertical ISB (V-EA) served as the reference for comparing the Horizontal ISB designs
Azevedo et al., 2024 [38]	In vitro	Full	Zirkonzahn White Scanmarker (PEEK) (Zirkonzahn GmbH)	Full arch connection	iTero Element 5D (Align) TRIOS 4 (3Shape) Primescan (Dentsply Sirona) Medit i700 (Medit Corp) Virtuo Vivo (Dental Wings)	Group cIOSs: Conventional digital scan technique (non-splinted scan bodies without landmarks)
Campana et al., 2024 [39]	Case Series clinical study	Full	Custom T-shape titanium scan bodies with lateral flags (LaStruttura, Digital Dentistry Solutions)	Full arch connection (modular units assembled chair-side)	TRIOS 4 (3Shape)	Optical impression taken without UST® in place (non-splinted)
Canullo et al., 2024 [40]	In vitro	Full	Sweden&Martina scan bodies	Full-arch (device fixed to scan body bases and length trimmed to fit)	iTero Element 5D (ITERO)<br><br>Trios 4 (3shape)<br><br>Carestream 3700 (CS)	Scans (ATOS compact Scan 5M) taken without the Auxiliary Geometric Device (no AGD group)
Chen et al., 2025 [41]	In vitro	Full	Not specified	Full arch	TRIOS 4 (3Shape A/S)	Intraoral scan alone (non-splinted/non-calibrated)
Cheng et al., 2024 [42]	In vitro	Full	IOS: Universal Scanbody (Segma).<br><br>MIOS (splinted): Multi Unit Scanbody (Segma).	Full arch	TRIOS 3 (3Shape)	CI: Conventional Impression (splinted with acrylic resin and scanned). IOS: Intraoral Scan without splinting. Baseline Reference: Industrial blue light scanner (ATOS Capsule 12m, ATOS).
Denneulin et al., 2023 [43]	In vitro	Full	Atlantis IO FLO (PEEK) (Dentsply Sirona)	Full arch connection (tied around scan bodies with supple knots)	Trios 3 (3Shape)<br><br>Primescan (Dentsply Sirona)	Non-splinted scan bodies. (Reference standard: Coordinate Measurement Machine - CMM)
Eddin and Önöral, 2024 [44]	In vitro	Full	Standard: CARES RC Mono Scan body (PEEK) (NucleOSS).<br><br>Custom: 3D printed custom scan bodies (CSB) with lateral extensions.	PR & CR: Full arch<br><br>AA & CSB: Lateral extensions	CEREC Omnicam (Dentsply Sirona)	IC: Conventional splinted open-tray impression (scanned cast).<br><br>SB: Intraoral scan without splinting (standard scan bodies).
Eid et al., 2024 [45]	In vitro	Full	CARES Monoscanbody (Institut Straumann AG)	Full arch	CEREC Primescan (Dentsply Sirona)	Group Cn: Conventional splinted open-tray impression (PVS/Flexceed) poured in stone and scanned with a lab scanner (E2; 3Shape)

St.Michael's
Inspired Care.
Inspiring Science.

Eldabe et al., 2025 [46]	In vivo prospective comparative study	Full	Group 1 (CSB): Conventional scan bodies (Neobiotech).<br><br>Group 2 (TMSB): Novel Tooth-Modified Scan Body (TMSB).	Non-continuous	AoralScan 3 (Shining3D)	Group 1 (CSB): Conventional scan bodies.<br><br>Reference: Verified conventional splinted open-tray impression scanned with a lab scanner).
Farah et al., 2025 [47]	In vitro	Full	CARES RC Mono Scanbody (PEEK) (Institut Straumann AG)	Non-continuous, Individual attachments placed on anterior scan bodies #13 and #23)	iTero Element 5D Plus (Align Technology)<br><br>CEREC Omnicam (Dentsply Sirona)	Scans without Geometric Attachments (GAs). (Reference standard: E4 desktop scanner).
Farahat and El Saaedi, 2025 [48]	In vitro	Full	Flotechno scan bodies (Milan, Italy)	Full arch connection	OVO (3Disc)	Group I: Conventional splinted open-tray impression (Gold standard).<br>Group II: Digital impression with separate scan bodies (Non-splinted).<br>D850 desktop scanner (3Shape).
Ferrini et al., 2024 [49]	In vitro	Full	PEEK abutments/scan bodies (Biosaf In s.r.l.)	Full arch connection	Medit i700 wired (Medit Corp)<br>Medit i700 wireless (Medit Corp)<br>CS3800 wireless (Carestream Health)<br>TRIOS 4 wireless (3Shape)	Reference scan obtained with a high-precision laboratory scanner (Alicona; Alicona Imaging GmbH).
Fu et al., 2024 [50]	In vivo clinical study	Full	Unique titanium scan bodies with a prefabricated bar splint	Non-continuous (Individual prefabricated bars connecting adjacent scan bodies)	TRIOS 3 (3Shape A/S)	OI: Open tray implant impression (Verified conventional impression scanned with lab scanner T8, Medit).<br>SPG: Stereophotogrammetry (PIC camera).
Fu et al., 2025 [51]	In vitro	Full	Unique titanium scan bodies with a prefabricated bar splint	Non-continuous (Individual prefabricated bars connecting adjacent scan bodies).	IOS-T: TRIOS 5 (3Shape)<br>IOS-M: Medit i700 (Medit)<br>IOS-A: Aoralscan 3 (Shining3D).	Without aid: Intraoral scanning of scan bodies without the prefabricated bar splints. (Reference: Laboratory scanner T8, Medit).
Garbacea et al., 2021 [52]	In vitro	Full	Core3dcentres® Scanbody (Nobel Biocare NobelActive RP Compatible)	Full arch	IOS: Trios 3 (3Shape)<br>True Definition (3M ESPE).<br>Lab/Industrial: Dental Wings 7 Series (DDW)<br>Atos Core 80 (GOM).	Unsplinted scan bodies scanned with each device. (Baseline reference: CMM - Coordinate Measuring Machine).
García-Martínez et al., 2022 [53]	In vitro	Full	Elos Accurate (IOS 2C-A) (Medtchech Pinol A/S)	Non-continuous (Individual rings attached to each scan body at varying heights to create irregularity).	CEREC Primescan (Dentsply Sirona)	UN Group: Unmodified scan bodies without rings. (Reference: CMM - Coordinate Measuring Machine).
Gianfreda et al., 2025 [25]	In vitro	Full	Control: Conventional PEEK scan	Non-continuous	Dexis 3800 (Dexis)	Conventional PEEK scan bodies without AGD. (Reference: Industrial structured light scanner ATOS compact Scan 5M).

			bodies (Nobel Biocare).<br><br>Test: Metallic L-shaped scan bodies with AGD (Nobel Biocare).			
Gómez-Polo et al., 2024 [54]	In vitro	Full	Avinent Transepithelial 4.8 scanbody (PEEK) (Avinent Implant System)	Full arch connection	TRIOS 3 (3Shape A/S)	IOS: Intraoral scan without splinting. (Reference: 7Series Desktop Scanner).
Huang et al., 2020 [55]	In vitro	Full	Group I (DO): Original scan body (Straumann).<br>Group II (DC): CAD/CAM scan body without extensional structure (Titanium alloy).<br>Group III (DCE): CAD/CAM scan body with extensional structure (Titanium alloy).	Non-continuous (Individual scan bodies with extensions)	TRIOS 3 (3Shape)	Group IV (CI): Conventional splinted open-tray impression (Silagum putty/light) scanned with a laboratory scanner (D2000).
Ileri et al., 2024 [56]	In vitro	Full	Bilimplant (Istanbul, Turkey)	gd1: Non-continuous (Individual tooth landmarks).<br>agd2: Full arch connection	Trios 3 (3Shape)<br>Medit i700 (Medit Corp)<br>Primescan (Dentsply Sirona)	std: Standard scanning without AGD.<br>CON: Conventional splinted open-tray impression (scanned with lab scanner).
Iturrate et al., 2019 [57]	In vitro	Full	Machined cylinders simulating scan bodies (part of the stainless-steel model)	Full arch connection	TRIOS 3 (3Shape)<br><br>True Definition (3M ESPE)<br><br>iTero Element (Align Technology)	Digital scan without the auxiliary geometric device (Reference: Industrial ATOS Compact Scan 5M/300 scanner).
Iturrate et al., 2019 [23]	In vitro	Full	Machined cylinders (integral to the model) simulating scan bodies	Full arch connection	TRIOS 3 (3Shape)<br><br>True Definition (3M ESPE)<br><br>iTero Element (Align Technology)	agdN Group: Digital scans of the edentulous model without the AGD. (Reference: Industrial ATOS Compact Scan 5M/300 scanner).
Kalayci et al., 2025 [58]	In vitro	Full	PEEK intraoral scan bodies (ISBs)	Non-continuous	Trios 3 (3Shape)	Group A: Single-stage scan (standard IOS).<br>Group B: Two-stage scan without additional reference markers.
Kanjanasavitree et al., 2022 [59]	In vitro	Full	PEEK scan bodies (Osstem Implant Co.)	FL Group: Full-arch connection.<br>LD/PIP: Non-splinted (Landmarks on ridge).	Trios 4 (3Shape)	CON: Unmodified master reference model (No landmarks/splinting). (Reference: E4 Lab Scanner).
Kao et al., 2023 [60]	In vitro	Partial	CARES Mono Scanbody 4.6 mm (Straumann)	Non-continuous	TRIOS 3 (3Shape)	Group A: Digital impression with scan bodies only (non-splinted). (Reference: E2 Desktop Scanner)
Ke et al., 2023 [61]	In vitro	Full	NB MU-R Scan body (Segma)	Non-continuous	TRIOS 4 (3Shape A/S)	Group IOS-NT/NA: Digital scans without landmarks.<br>Group CNV: Conventional splinted open-tray impression (scanned with lab scanner)

					Aoralscan 3 (Shining 3D	
Kernen et al., 2022 [62]	In vitro	Full	**Original intraoral scan bodies (Camlog)**	Full arch connection	CS3600 (Carestream Dental LLC)	Unsplinted scans (standard ISBs without aid). Reference: Desktop optical scanner (E3, 3Shape)
Lam et al., 2025 [63]	In vitro	Full	Neodent scan bodies (Straumann)	Full arch connection	Primescan (Dentsply Sirona)	Group IOS: Intraoral scanning without AGP (Non-splinted)<br><br>Group OTS: Open-tray splinted conventional technique<br>Reference: Industrial scanner (ATOS Core 200)
Laureti et al., 2025 [64]	In vitro	Full	Horizontal (H-ISBs): Multi-unit Polo (H-NB), Nexus Scan Gauges (H-NS), M6 Dental (H-M6), SmartFlags (H-SF).<br><br>Vertical (V-ISB): Elos Accurate (V-EA)	Not applicable	iTero Element 5D (Align Technology)<br><br>Primescan (Dentsply Sirona)<br><br>TRIOS 3 (3Shape)<br><br>TRIOS 4 (3Shape)	V-EA (Vertical ISB) served as the comparison group
Li et al., 2024 [65]	In vitro	Full	Group IOS-C: CARES Mono Scanbody (Institute Straumann AG)<br><br>Group IOS-M: Modified scan bodies (Digital Wings; Segma Medical Technology)	Non-continuous	TRIOS 3 (3Shape A/S)	Group IOS-C: Conventional scan bodies.<br><br>Group CNV: Conventional open-tray splinted impression (dental floss and Pattern Resin)
Li et al., 2024 [66]	In vitro	Full	Straumann intraoral scan bodies	Full arch connection	TRIOS 3 (3Shape A/S)	Group IOS: Intraoral scan only (no aid).<br>Group CONV: Conventional splinted open-tray impression<br>Reference: Desktop scanner (LS3, Kavo)
Liu et al., 2025 [67]	In vitro	Full	Original scanning rods	Non-continuous	TRIOS 4 (3Shape)	Intraoral scan without auxiliary devices (labeled "B).<br>Reference: Laboratory scanner (D2000, 3Shape)
Liu et al., 2024 [68]	In vitro	Full	Bridge+; Segma Medical Devices Corp	Non-continuous	TRIOS 3 (3Shape A/S)	Group OI: Open-tray impression splinted with metal bars and Pattern Resin (scanned with lab scanner).<br>Group SPG: Stereophotogrammetry (PIC camera) using photogrammetric transfers.<br>Reference: Medit T8 lab scanner.
Lu et al., 2025 [69]	In vitro and In vivo	Full	Cylindrical scan bodies (SEGMA Tech)	Non-continuous	iTero Element 5D Plus (IT)<br>Medit i500 (MD)<br>TRIOS 5 (TR)	Conventional splinted open tray impression (CI)<br>laboratory<br>scanner (E4; 3Shape A/S)
Lyu et al., 2025 [70]	In vitro	Full	SB-M (SEGMA Corp.)	Non-continuous	,TRIOS Scanner 3 (3Shape A/S)	Group IOS: Digital scan only (no aid).<br>Group CI: Conventional splinted open-tray impression.<br>Reference: 3Shape D2000 Lab Scanner.
Masu et al., 2021 [71]	In vitro	Full	Scan bodies (Position Locator Multiple Nobel Biocare Multi-unit Abutment, Nobel Biocare)	Full arch connection	Primescan (Dentslpy Sirona)<br><br>TRIOS 3 (3Shape A/S)<br><br>3M True Definition (3M ESPE)	Type 0: Digital scan without aid. CON: Conventional silicone impression (splinted)<br><br>VJ (Verification Jig) —analog reference<br><br>3Shape D810 lab scanner
Mizumoto et al., 2020 [72]	In vitro	Full	AF (IO-Flo, Dentsply),<br><br>NT (Nt-Trading)<br><br>DE (DESS)	GB, PP non-continuous<br><br>FL full arch connection	TRIOS (3Shape)	NO Group: Unmodified master model (Non-splinted/No aid).<br><br>Reference: Industrial blue light scanner (COMET L3D)

			C3D (Core3Dcentres)<br><br>ZI (Zimmer Biomet)			
Nedelcu et al., 2023 [73]	In vivo clinical study	Full	Elos Accurate IO 6A-B (Elos Medtech)	Full arch connection	TRIOS 3 (3Shape)	Group CT: Control scan (non-splinted/no aid)<br><br>Reference: In situ scan using an industrial scanner (ATOS Core 80)
Nulty et al., 2024 [74]	In vivo retrospective pilot study	Full	IPD scan bodies	1. non-continuous (2 implants connected)<br>2. Full arch connection	Primescan (Dentsply Sirona)<br><br>Medit i900	Scanner without aids (Traditional scanning).<br><br>Reference: Master STL from Ineos X5 lab scanner (from impression)
Önöral and Çakır, 2024 [75]	In vitro	Full	NucleOSS scan bodies (TO 32033)	Non-continuous	CEREC Omnicam (Dentsply Sirona)	Reference: Benchtop extraoral scanner (inEOS X5)
Önöral et al., 2025 [76]	In vitro	Partial	NucleOSS scan bodies (TO 32033)	Non-continuous	CEREC Omnicam (Dentsply Sirona)<br><br>iTero Element 5D Plus (Align Technology)	Digital scan without PAD (No PAD group).<br><br>Reference: Benchtop lab scanner (inEOS X5)
Pan et al., 2021 [77]	In vitro	Full	PEEK scan bodies (Zfx intrascan matchholder H4, Zimmer Biomet)	Full arch connection	TRIOS 3 (3Shape A/S)	Group 0: Simulated mucosa (silicone) without auxiliary device.<br><br>Reference: Laboratory scanner (IScan D104i)
Pereira et al., 2022 [78]	In vivo clinical study	Full	Scan bodies (Neodent)	Full arch connection	TRIOS (3Shape)	Group SC: Intraoral scanning with scan bodies only.<br><br>Group CI: Conventional impression (Solid Index + Open Tray)
Pereira et al., 2022 [79]	In vivo clinical study	Full	Neodent scan bodies (Institut Straumann AG)	Full arch connection	TRIOS (3Shape A/S)	Group SC: Intraoral scanning with scan bodies only.<br><br>Group CT: Conventional splinted impression (poured and scanned with lab scanner)
Pereira et al., 2022 [80]	In vitro	Full	Neodent scan bodies	Full arch connection	TRIOS (3Shape A/S) [Intraoral - SI]<br><br>S600 ARTI Scan (Zirkonzahn) [Extraoral - SE]	Group SB: Scan bodies only
Pol et al., 2024 [81]	In vitro	Full	Not specified	Full arch connection	TRIOS 3 (3Shape A/S)	Reference scan A1 (Digital without splinting) and B1 (Conventional without splinting)
Pozzi et al., 2022 [82]	In vitro	Full	PEEK scan bodies (LaStruttura spa)	Full arch connection	TRIOS 3 (3Shape A/S)	Group ISS-: Digital impression without splinting (Control).<br><br>Reference: Industrial structured blue light scanner (ATOS Compact Scan 5M)
Retana et al., 2023 [83]	In vitro	Full	ISBs (conical connection L10mm Scan Post;MIS Implant Technologies)	Full arch connection (Splinted model)	Cerec Primescan<br><br>Cerec Omnicam<br><br>Trios 4<br><br>Trios 3<br><br>Planmeca Emerald<br><br>Medit i500<br><br>Carestream 3600.	Control Group: Non-splinted scans with the same 7 IOSs.<br><br>Reference: Extraoral laboratory scanner (InEos X5).
Revilla-León et al., 2025 [84]	In vitro	Full	Standard subgroup: Standard ISBs (Accurate Implant Body	Non-continuous	TRIOS 5 (3Shape A/S)	Stand Subgroup: Nonsplinting technique with standard ISBs.<br><br>Reference: Calibrated laboratory scanner (T710, Medit Corp.).

St. Michael's
Inspired Care.
Inspiring Science.

			MUA, Elos Medtech). Apollo subgroup:<br><br>Horizontal noncalibrated ISBs (Apollo, Apollo) which are single- or dual-wing devices.		Medit i700 (Medit Corp.)<br><br>Primescan (Dentsply Sirona)<br><br>Aoralscan 3 (Shining 3D)<br><br>iTero Element 5D Plus (Align Technologies)	
Revilla-León et al., 2025 [85]	In vitro	Full	NCS-IOC subgroup: Noncalibrated splinting horizontal ISBs (IOConnect; TruAbutment) which connect in the center of the palate.	Non-continuous	TRIOS 5 (3Shape A/S)<br><br>Medit i700 (Medit Corp.)<br><br>Primescan (Dentsply Sirona)<br><br>Aoralscan 3 (Shining 3D)<br><br>iTero Element 5D Plus (Align Technologies)	NS-ISB subgroup: Nonsplinting technique with standard ISBs (TrueScan Body).<br><br>Reference: Calibrated laboratory scanner (T710, Medit).
Revilla-León et al., 2025 [86]	In vitro	Full	Groups tested:<br><br>Nonconnected ISBs (ISB): Standard ISBs (Scan Abutment Non-Engaging IPD).<br><br>Splinted ISBs (SSB): 3D printed framework connected with Pattern Resin.<br><br>Calibrated Framework (CF): Calibrated metal framework (IOSFix) connected with Pattern Resin.<br><br>Photogrammetry (PG): Optical markers (PIC Transfer)	Full arch connection	TRIOS 4 (3Shape A/S)<br><br>Medit i700 (Medit Corp.)<br><br>iTero Element 5D Plus (Align Technologies)<br><br>CS3800 (Carestream Dental)	Group LBS: Laboratory scanner (T710) with same subgroups.<br><br>Reference: Coordinate Measurement Machine (CMM).
Revilla-León et al., 2025 [87]	In vivo clinical study	Full	IOConnect: Noncalibrated horizontal splinting ISB (TruAbutment)<br><br>Elite: Intraoral photogrammetry marker (Shining 3D)<br><br>MicronMapper: Snap-on extraoral PG	Non-continuous	TRIOS 5 (3Shape A/S) for IOConnect<br><br>Aoralscan Elite (Shining 3D) for Elite group.<br><br>MicronMapper (SIN	Reference: Extraoral PG system with screw-retained markers (Micron Mapper).

St. Michael's
Inspired Care.
Inspiring Science.

			markers (SIN Dental)		Dental) for Extraoral PG.	
Roig et al., 2022 [88]	In vivo clinical study	Full	Scan bodies (Core3D)	Full arch connection	TRIOS 3 (3Shape A/S)	Group CF: Conventional impression (Open tray with splinted copings). Reference for fit comparison
Rustichini et al., 2025 [89]	In vivo Retrospective clinical study	Full	Not specified	Full arch connection	CS 3600 (Carestream Dental)	Not applicable (Descriptive study of the CSF protocol outcomes)
Rutkunas et al., 2022 [90]	In vitro	Not applicable	Modified impression copings with scan bodies (CARES RC Mono Scanbody) attached on top	Connecting two adjacent impression copings	Laboratory scanner (E4, 3Shape)	Unsplinted impression copings (baseline).<br><br>Reference: E4 Lab scanner scans of the unsplinted copings
Tallarico et al., 2020 [91]	In vitro	Full	Built-in scan abutments	Full arch	Medit i500 (Medit Corp)	Digital impression taken without the prosthetic-based impression template
Wu et al., 2024 [92]	In vitro	Full	IO 2C-A (ELOS MEDTECH)	Non-continuous	Aoralscan 3 (Shining3D)	Scan without the auxiliary devices<br><br>Reference scan with D2000 (3Shape)
Wu et al., 2024 [93]	In vitro	Full	IO 2C-A (ELOS MEDTECH)	Non-continuous	Aoralscan 3 (Shining3D)	Group I: Scanning without any artificial landmarks<br><br>Conventional open-tray impression<br><br>Reference scan with D2000 (3Shape)
Wu et al., 2024 [94]	In vitro	Full	IO 2C-A (ELOS MEDTECH)	Non-continuous	Aoralscan 3 (Shining3D)	Scanning performed without the PAD<br><br>Reference scan with D2000 (3Shape A/S)
Wu et al., 2023 [95]	In vitro	Full	IO 2C-A (ELOS MEDTECH)	Full arch connection	Aoralscan 3 (Shining3D)	Group A (CO): Scanning performed without any SBCs<br><br>Reference scan with D2000 (3Shape)
Kurtulmus-Yilmaz et al., 2025 [96]	In vitro	Full	Elos Accurate Scan Body IO 2C-A (PEEK material)	Non-continuous	iTero Element 5D Plus (Align Technology)	Scanning performed without using scan aids<br><br>Reference scan with inEOS X5 (Dentsply Sirona)
Zhang et al., 2024 [97]	In vitro	Full	Group OS: Original CARES Mono Scan body (Straumann)<br><br>Groups CS, CSS, CSA: Custom CAD/CAM Grade 5 titanium alloy scan bodies (sandblasted)	Non-continuous (extensions designed to be adjacent to each other)	TRIOS 3 (3Shape)	Group CI: Conventional splinted open-tray impressions (Silicone)<br>Original CARES Mono Scan Body<br>Reference scan with laboratory scanner (D2000, 3Shape)
